# Editorial for the Special Issue “Gels for Removal and Adsorption (3rd Edition)”

**DOI:** 10.3390/gels12050400

**Published:** 2026-05-06

**Authors:** Zhenxing Fang, Daxin Liang, Shiyang Li

**Affiliations:** 1 Ningbo Key Laboratory of Agricultural Germplasm Resources Mining and Environmental Regulation, College of Science and Technology, Ningbo University, Ningbo 315300, China; 2 College of Materials Science and Engineering, Northeast Forestry University, Harbin 150040, China; 3 Key Laboratory of Organic Compound Pollution Control Engineering (MOE), School of Environmental and Chemical Engineering, Shanghai University, Shanghai 200444, China

## 1. Introduction

In the various fields of materials science, functional gel materials stand out as a rapidly emerging and increasingly prominent field [1,2,3,4]. These soft-matter systems, composed of three-dimensional polymer networks swollen with a large amount of solvent, have captivated the attention of researchers worldwide, owing to their unique physicochemical properties and broad application prospects. Evolving from objects of fundamental chemical research to indispensable components in diverse critical fields such as biomedicine, environmental engineering, flexible electronics, and energy science, the development of functional gel materials represents a history of innovation, progressing from theory to practice and from the laboratory to industrialization.

The fascination of functional gel materials stems from the high degree of designability in their structure and properties. By precisely tailoring the chemical composition, cross-linking density, and network architecture of polymers and incorporating functional nanofillers or bioactive molecules, scientists can endow gel materials with a remarkable array of characteristics. For instance, they can be as soft, moist, and biocompatible as biological tissues, making them ideal scaffolds for tissue engineering and carriers for drug delivery [5]. They can respond to external stimuli such as temperature, pH, light, or specific molecules, enabling intelligent substance release or shape memory effects. Furthermore, they can be engineered to possess ultrahigh adsorption capacities, excellent electrical conductivity, or efficient catalytic activity. This “molecular building block” capability allows functional gel materials to precisely meet the demands of various application scenarios, positioning them as key materials for addressing major 21st-century challenges in the environment, energy, and health [6,7,8].

## 2. Overview of the Publications in This Special Issue

The nine research papers carefully selected for this issue vividly exemplify the cutting-edge explorations in the field of functional gel materials. These contributions span multiple levels, from fundamental scientific questions to practical application technologies, not only deepening our understanding of gel formation mechanisms, structural evolution laws, and performance modulation strategies but also providing highly valuable new ideas and methods for functional gel materials to solve real-world problems.

In the critical field of environmental remediation and separation technology, which is vital to sustainable human development, functional gel materials have demonstrated exceptional application potential. With the rapid development of industry and continuous population growth, water pollution has become an increasingly severe global issue, creating an urgent demand for efficient, environmentally friendly, and cost-effective water treatment technologies. Several papers in this issue focus on this area, offering innovative solutions. For example, addressing the global challenge of heavy metal ion pollution in water, a novel composite adsorbent gel was developed by combining algae biomass with alginate. Algae, as natural biosorbents, possess cell walls rich in hydroxyl, carboxyl, and other functional groups that exhibit a high affinity for heavy metal ions. By immobilizing algae within the three-dimensional network of alginate hydrogels, the researchers not only preserved the adsorption activity of the algae but also significantly enhanced the mechanical stability and recyclability of the material. Experimental results showed that the composite hydrogel’s adsorption capacities for various heavy metal ions, such as Cu^2+^, Cr^3+^, and Co^2+^, were increased by 38.0%, 20.6%, and 27.1%, respectively, compared to pure alginate hydrogels. This study not only provides an efficient material option for the remediation of heavy-metal-contaminated water but also opens up a new avenue for the high-value utilization of algae biomass.

Another study focused on the treatment of organic dye wastewater. Methylene blue (MB) is a cationic dye widely used in textile, paper, and other industries, and its discharge poses a serious threat to aquatic ecosystems and human health. Thus, nanocellulose and polyacrylamide were used to synthesize an interpenetrating network gel via a one-step method, which was then chemically modified by NaOH. The modified gel not only possesses a rich porous structure and numerous adsorption sites but also achieves a maximum adsorption capacity of 172.08 mg/g for methylene blue. More importantly, the gel exhibited excellent reusability, maintaining an adsorption efficiency of over 85% after four adsorption–desorption cycles. Additionally, the MB-saturated gel was used as a controlled-release fungicide for fish, achieving “waste-to-wealth” transformation and multifunctional utilization of the material, and providing a forward-thinking approach to solving the problem of adsorbent secondary pollution.

In the fields of biopharmaceuticals and protein engineering, the removal of endotoxins (lipopolysaccharides, LPS) is a crucial yet highly challenging step. Even at extremely low concentrations, endotoxins can trigger severe physiological reactions such as fever and shock in the human body, necessitating their complete removal from protein drugs and biological products. Traditional separation methods often suffer from low efficiency, loss or denaturation of target proteins, and other issues. To address this challenge, researchers designed and prepared a macroporous cryogel functionalized with polymyxin B (PMB). PMB is a cationic cyclic polypeptide antibiotic that can specifically bind to the lipid A moiety of endotoxins through electrostatic and hydrophobic interactions. By covalently conjugating PMB to the macroporous cryogel backbone, the material not only possesses an extremely high endotoxin adsorption capacity (up to 1408.38 EU/mg) but also allows high-concentration protein solutions to flow through rapidly due to its open macroporous structure, effectively avoiding the clogging problems associated with traditional fillers. Experimental results show that in complex systems containing multiple high-concentration proteins (e.g., BSA, HSA, and Hb), the functionalized cryogel still achieved an endotoxin removal efficiency of up to 99.62% while maintaining a high recovery rate of target proteins. This work provides a novel and highly promising technical platform for efficient and highly selective endotoxin removal in the biopharmaceutical industry.

Moreover, an investigation into the behavior of polyacrylamide (PAM) gel in underwater cement slurry offered significant theoretical points for its utilization in the field of construction materials. This study systematically elucidated the microscopic flocculation mechanism of PAM in cement pastes with a high dosage of polycarboxylate ether (PCE) superplasticizer. Various techniques, including zeta potential measurements, Fourier transform infrared spectroscopy, and total organic carbon analysis, were employed to propose a three-stage “adsorption–lubrication–entanglement” model. The rheological properties of cement-based materials were illustrated at a microscopic level through this three-stage model, establishing a scientific foundation for their precise application in practical applications, including anti-washout and thickening of underwater concrete.

Functional gel materials are emerging as key components for the next generation of wearable technology and human–machine interaction interfaces in the cutting-edge fields of flexible electronics and intelligent sensing, which are driving the advancement of science and technology. Traditional rigid electronic devices result in poor comfort and limited movement when in contact with human skin, whereas flexible sensors based on conductive hydrogels perfectly address these pain points.

One review article in this issue systematically and comprehensively summarizes the latest research progress in conductive hydrogel-based flexible wearable sensors for sports monitoring applications. The review first introduces the key characteristics of conductive hydrogels, including the sources of their conductivity (ionic, electronic, or mixed), self-adhesion, self-healing capabilities, and excellent biocompatibility. It then analyzes the working principles of various sensing mechanisms in sports monitoring. On this basis, the review focuses on the latest methods for modifying conductive hydrogels to enhance their mechanical strength, environmental stability (e.g., freeze resistance and anti-drying), and sensing performance (e.g., sensitivity and response speed) through strategies such as polymer blending, polyelectrolyte doping, inorganic salt introduction, and nanomaterial composite. Finally, this article presents prospects on the application potential of conductive hydrogel flexible sensors in capturing human motion posture, electrophysiological signal detection, and biochemical indicator analysis, and points out the current challenges, such as long-term stability, multi-signal integration, and combination with artificial intelligence algorithms. This review provides valuable reference materials and a clear development roadmap for researchers in related fields.

Another original study reports a high-performance flexible sensing hydrogel with a hierarchical network structure. By introducing graphene oxide (GO) and glycerol into a nanocellulose/sodium alginate/polyacrylamide composite system via a one-step method, the researchers successfully constructed a hierarchical network hydrogel with excellent structural stability, freeze resistance, anti-drying properties, and high sensitivity. Experimental results showed that the hydrogel could maintain structural integrity after 100 compression cycles; after drying at 30 °C for 24 h, its mass retention rate reached 48%, and its melting peak was as low as −13.87 °C, indicating that it could maintain good flexibility even in low-temperature environments. More importantly, the introduction of GO regulated the arrangement of the hydrogel network through steric hindrance and electrostatic interactions, enabling it to exhibit excellent strain-sensing performance. Interestingly, the research team proposed using it as a form of smart dressing to absorb heat from the skin’s surface due to its good thermal conductivity, thereby alleviating joint and muscle discomfort caused by strenuous exercise. 

In the field of energy and catalytic applications, functional gel materials also show great application potential, providing new material platforms for solving the energy crisis and environmental problems.

With the global pursuit of carbon neutrality goals, how to efficiently capture and convert carbon dioxide (CO_2_) has become a research hotspot. Photocatalytic CO_2_ reduction technology, which uses solar energy to convert CO_2_ into high-value-added fuels such as methane and methanol, is considered a highly promising green technology route. Two studies in this issue focus on this field and adopt innovative gel material strategies.

One study reports a Cu_9_S_5_/gel-derived TiO_2_ composite synthesized via a hydrazine-hydrate-assisted hydrothermal method. The composite obtained had a rich pore structure and high specific surface area, providing sufficient sites for CO_2_ adsorption and activation. Experimental results showed that the optimized composite exhibited excellent performance in the photocatalytic CO_2_ reduction reaction. The CO_2_ conversion performance was dramatically increased with a methane production rate of 34 μmol·g^−1^·h^−1^ and a methane selectivity of up to 64.76%. Density functional theory (DFT) calculations further revealed that the Cu_9_S_5_/TiO_2_ interface can effectively reduce the formation energy barriers of key reaction intermediates, thereby promoting the efficient adsorption of CO_2_ and its conversion to methane. This work provides a new strategy for designing efficient integrated photocatalysts for CO_2_ adsorption and conversion.

Another related study successfully introduced sulfur vacancies into a ZnS/gel-derived TiO_2_ heterojunction via hydrazine treatment. The sulfur vacancies not only provide more active sites for the adsorption and activation of CO_2_ but also promote the separation and transfer of photogenerated charges by regulating the interfacial electronic structure. Mott–Schottky analysis and optical bandgap measurements indicated significant interfacial charge redistribution in the composite, forming a charge migration pathway similar to a Z-scheme heterojunction, which retains electrons with strong reducing power and holes with strong oxidizing ability for CO_2_ reduction and water oxidation, respectively. Experimental results showed that both the methane yield and selectivity of the catalyst significantly improved. This study reveals the crucial role of defect engineering in regulating the performance of photocatalysts and provides important theoretical guidance for designing highly selective photocatalysts for CO_2_ reduction.

In addition, in the field of solar energy utilization, achieving efficient solar steam generation using functional gel materials has become a research hotspot in recent years. Traditional solar distillation technologies suffer from low energy conversion efficiency, whereas interfacial solar steam generation (ISSG) technology, based on photothermal conversion materials, achieves efficient heat utilization by localizing photothermal materials at the gas–liquid interface. One study in this issue incorporated oxygen-deficient tungsten oxide (W_18_O_49_) nanowires with excellent near-infrared light absorption and photothermal conversion properties into a polyvinyl alcohol (PVA) gel network, successfully preparing a high-performance composite photothermal gel. This study first synthesized W_18_O_49_ nanowires rich in oxygen vacancies by treating commercial WO_3_ at a high temperature in a nitrogen atmosphere (lattice oxygen escaping method). Then, it was mixed with a PVA solution and cross-linked by glutaraldehyde to prepare a composite gel with a three-dimensional network structure. The hydrophilicity of the PVA hydrogel ensures rapid water transport to the evaporation interface, while its low thermal conductivity effectively inhibits heat loss to the bulk water. Experimental results showed that the solar steam generation rate of the W_18_O_49_@PVA composite gel was as high as 2.65 kg·m^−2^·h^−1^, which is much higher than that of pure PVA gel, providing a simple and low-cost solution for efficient seawater desalination and wastewater treatment using solar energy.

## 3. Conclusions

In conclusion, the nine research papers featured in this issue comprehensively demonstrate the cutting-edge applications and innovative achievements of functional gel materials in addressing critical global challenges across fields such as energy, electronics, and the environment. These studies have not only advanced material design, synthetic strategies, and performance control but also accelerated the translation of functional gel materials from laboratory research to practical implementation. We believe that with the continuous progress of scientific research and technological development, functional gel materials will undoubtedly play an increasingly important role in more industries and make distinctive contributions to building a smarter, greener, and more sustainable future.
